# Metabonomic study of the protective effect of Fukeqianjin formula on multi-pathogen induced pelvic inflammatory disease in rats

**DOI:** 10.1186/s13020-018-0217-6

**Published:** 2018-12-10

**Authors:** Yan Zhang, Wei Li, Liang Zou, Yun Gong, Peng Zhang, Shasha Xing, Hang Yang

**Affiliations:** 1 School of Medicine, Chengdu University, No. 2025, Cheng Luo Road, Chengdu, 610106 Sichuan People’s Republic of China; 2 Zhuzhou Qianjin Pharmaceutical Ltd. Co., No. 801 Zhuzhou Avenue, Tianyuan District, Zhuzhou, 412000 Hunan People’s Republic of China; 3 Drug Clinical Trial Center, Affiliated Hospital of Chengdu University, 2nd Ring Road, Jinniu District, Chengdu, 610081 Sichuan People’s Republic of China

**Keywords:** Fukeqianjin formula, Anti-inflammatory, UPLC-Q-Exactive Orbitrap-MS, Metabonomic, Pelvic inflammatory disease

## Abstract

**Background:**

Fukeqianjin formula has been effectively used in the treatment of pelvic inflammatory disease (PID) and the related complications in clinic. Although there have been some studies about the underlying mechanism that focus on its anti-inflammatory and immunoregulatory activities. But the mechanism is still not fully understood. The aim of this study was to investigate the alteration of plasma metabolic profiles in PID rats and the regulatory effect of Fukeqianjin formula on potential biomarkers.

**Methods:**

Pelvic inflammatory model was established by intrauterine inoculation of multiple pathogens combined with mechanical injury of endometrium. Rats were randomly divided into normal group, model group, azithromycin group, high-and low-dose of Fukeqianjin formula treatment group (FF-H, and FF-L, respectively). After 14 days of intragastric administration, the plasm levels of interleukin-1β (IL-1β) and nitric oxide (NO) were measured. To further recognize and identify potential biomarkers and metabolic pathways, an ultra-performance liquid chromatography-quadrupole-Exactive Orbitrap-mass spectrometry (UPLC-Q-Exactive Orbitrap-MS) metabonomic method combined with multivariate analyses including principal component analysis (PCA), partial least squares discriminant analysis (PLS-DA) and orthogonal partial least squares discriminant analysis (OPLS-DA), was employed to analyze the metabolic profiling.

**Results:**

Compared with normal group, the plasma levels of IL-1β and NO were significantly increased in the PID model group (P < 0. 05), and obviously decreased after high-dose intervention of Fukeqianjin formula (P < 0. 01). The PCA, PLS-DA and OPLS-DA analysis showed that PID rats were clearly separated from normal rats. Compared with the PID model group, the metabolite profiles of Fukeqianjin formula treatment group was gradually restored to normal. Meanwhile, 14 potential metabolite biomarkers, which were mainly related to the metabolic pathways of intervening glycerophospholipid metabolism, linoleic acid metabolism/alpha-linolenic acid metabolism, amino acid metabolism, arachidonic acid metabolism, and unsaturated fatty acids biosynthesis, have been identified. Fukeqianjin formula exerts good regulatory effect on the abnormal metabolism of PID rats.

**Conclusions:**

Intrauterine inoculation of multiple pathogens combined with mechanical injury of endometrium could significantly disturb the plasma metabolic profiles of rats. Fukeqianjin formula has potential therapeutic effect on multi-pathogen-induced PID by ameliorating metabolism disorders and alleviating the inflammatory response.

**Electronic supplementary material:**

The online version of this article (10.1186/s13020-018-0217-6) contains supplementary material, which is available to authorized users.

## Background

Pelvic inflammatory disease (PID) is a multiple bacterial infection and inflammatory disorder on the upper female genital tract, typically involving the uterus, fallopian tubes, and ovaries [1]. The widespread gynecologic disease, which can lead to serious sequelae, such as tubal infertility, ectopic pregnancy, and chronic pelvic pain, is considered to be a major threat to reproductive age women [2]. Studies based on the updated meta-analysis have also demonstrated that PID might be a potential risk factor of ovarian cancer, especially among Asian women [3]. The most common pathogens cause to PID were *Neisseria gonorrhoeae*, *Chlamydia trachomatis*, *Mycoplasma genitalium*, and gram-negative bacilli [4]. In addition, studies confirm that *Staphylococcus aureus*, *Escherichia coli*, and *S. sanguinegens* were also associated with PID [5, 6]. Currently, PID is commonly treated with broad spectrum antibiotics. A randomized clinical trial confirmed that azithromycin monotherapy or azithromycin monotherapy in combination with metronidazole has an excellent therapeutic effect [7]. Moreover, several antibiotics including ceftriaxone, cefoxitin, doxycycline, and metronidazole also provide a good antibacterial efficacy and high clinical cure rate on pelvic inflammation [8, 9].

In recent years, accumulating evidence suggests that Chinese medicine has a remarkable curative effect in the treatment of PID. Research confirms that apart from the infection caused by pathogenic microorganisms, long-term chronic inflammation can also result in the decline of the immune system or pelvic microcirculation disturbance [10], while traditional Chinese medicine has various functions including anti-bacterial, anti-inflammatory, regulating immunity, or promoting blood circulation. An experimental study has demonstrated that *Patrinia villosa* can provide a good therapeutic effect on pelvic inflammatory rats by significantly reduce the levels of pro-inflammatory cytokines such as IL-6, IL-8 and TNF-α in serum [11]. Studies have shown that Danzhi Decoction not only has a positive anti-inflammatory effect, but also can relieve chronic pelvic pain and ameliorate the pelvic microcirculation disorders in PID mice [12]. The laboratory study confirms that *Cortex phellodendri* and *Humulus japonicus* exert good therapeutic effect on mice with chronic PID by inhibiting the expression of inflammatory cytokines and neutrophil infiltration [13]. Furthermore, Feiyangchangweiyan is reported to suppress infiltration and apoptosis of inflammatory cells in uterine tissues by preventing NF-κB nuclear translocation in pathogen-induced PID rats [14].

Fukeqianjin formula, consisting of *Moghania macrophylla*, *Radix Rosa laevigata*, *Andrographis paniculata*, *Mahonia fortunei*, *Zanthoxylum dissitum hemsl*, *Angelica sinensis*, *Spatholobus suberectus*, and *Codonopsis pilosula*, is a traditional Chinese medicine prescription which has been widely applied to treat various gynecological inflammation disease clinically in China. Previous studies have verified that Fukeqianjin tablet could improve the immune function by promoting the production of IgA, IgG, and IgM in acute PID rats [15]. Furthermore, the experimental study confirmed that the anti-inflammation mechanism of Fukeqianjin tablets might be associated with reducing level of inflammatory cytokines IL-1β, IL-8 and TNF-α, and down-regulating of the expression of TNF-α, IL-10, and IL-2 mRNA [16, 17]. Additionally, Fukeqianjin tablets can improve the expression of caspases-3 and caspases-8, mediate inflammatory cells apoptosis, and ultimately alleviate pathological damage of ovarian tissue [18].

As an important branch of systematic biology, metabolomics is mainly used to evaluate the effects of environment, disease status, or drug intervention on endogenous small molecular metabolites like fatty acids, amino acids, peptides, and lipids [19]. Recently, metabolomic investigation has been widely used in the evaluation of biological efficacy and possible mechanism of traditional Chinese medicine. An GC/MS-based metabolomics research revealed that the metabolic disorders of citric acid circulation, glucose metabolism, unsaturated fatty acids and amino acids in acute liver injury mouse can be regulated by *Hedyotis diffusa* [20]. A recent metabolomic study suggested that 12 biomarkers were identified in carbon tetrachloride (CCl4)-induced liver fibrosis in rats, and Shu Gan Jian Pi formula could ameliorate the energy, amino acid, sphingolipid, cytochrome P450, glucose and water–electrolyte metabolism [21]. Meanwhile, PID-associated metabolomic study in the urine of PID rats based on GC–MS has yet been reported, eighteen potential biomarkers of PID were found, and *Patrinia scabiosaefolia Fisch* showed a holistic interventional effect on disease-associated metabolomic changes [22, 23].

In the present study, the effect of Fukeqianjin formula on inflammatory cytokines was investigated in PID rats induced by intrauterine inoculation of multiple pathogens combined with mechanical injury of endometrium. Moreover, an UPLC-Q-Exactive Orbitrap-MS-based metabonomic method combined with multivariate analyses including principal component analysis (PCA), partial least squares discriminant analysis (PLS-DA) and orthogonal partial least squares discriminant analysis (OPLS-DA), were used to analyze the metabolic profiling and to recognize and identify potential biomarkers and the metabolic pathways.

## Methods

### Information of experimental design and resources

The information regarding the experimental design, statistics, and resources used in this study are attached in the minimum standards of reporting checklist (Additional file 1).

### Chemicals and reagents

Ferulic acid (Batch No. 110773-200611) was obtained from the National Institutes for Food and Drug Control (Beijing, China). Chlorogenic acid (Batch No. MUST-17030620) was obtained from Chengdu Manchester biotech Co., Ltd. (Chengdu, China). Lobetyolin (Batch No. 16022204), Andrographolide (Batch No. 16073103), and dehydrated andrographolide (Batch No. 16022501) were obtained from Chengdu Zhuo Pu Instrument Co., Ltd (Chengdu, China). Fukeqianjin tablets were obtained from Zhuzhou Qianjin pharmaceutical Co., Ltd. (Hunan, China). Azithromycin (Batch No. R37977) was purchased from Pfizer pharmaceutical Co., Ltd. (NY, USA). *Staphylococcus aureus* (ATCC25923) and *E. coli* were purchased from Chinese national fungus storehouse. Methanol and acetonitrile with HPLC-grade were purchased from Thermo Fisher Scientific Inc. (Iowa, USA). Formic acid was purchased from Sigma Chemical Co. (St. Louis, MO, USA). Water used in this study was prepared by ULUP Ultrapure Water System (Chengdu, China).

### Animals

Healthy female Sprague–Dawley rats (specific pathogen-free, 220 ± 20 g) were supplied by DaShuo Biotechnology Co., Ltd [Approval Number: SCXK (Sichuan) 2015-030, Chengdu, China]. This study was strictly in accordance with the Guidelines for the Care and Use of Laboratory Animals. The animal protocol was approved by the Animal Ethics Committee of Chengdu University.

### Preparation and quality control of Fukeqianjin formula

Fukeqianjin formula powder (4 g) was extracted with 20 mL 70% ethanol by ultrasonic extraction for 60 min. Then the supernatant was concentrated and dissolved with 5 mL methanol after centrifugation for 10 min at 12,000 rpm. Samples for High Performance Liquid Chromatography (HPLC) detection were obtained by filtration through a 0.22 μm membrane filter.

A HPLC method was established for the identification of the major compounds in Fukeqianjin formula (LC-20AT HPLC system, Shimadzu, Japan). A ZORBAX SB-C18 analytical column (4.6 × 250 mm, 5 μm, Agilent, USA) was used with the column temperature maintained at 35 °C. Acetonitrile and 0.1% phosphoric acid in water were treated as mobile phase A and B, respectively. The gradient elution was programmed as follows: 10% A–20% (0–15 min), 20–47.5% A (15.1–45 min), 47.5–68.5% A (45.1–50 min). The detection wavelength is 220 nm. The flow rate was set at 1.0 mL/min, and the sample injection volume was set at 5 μL.

### Induction of PID model rats

The preparation of PID model was based on the methods described in the previous literature with some revisions [23]. Rats were anesthetized with 20% urethane (5 mL/kg) by intraperitoneal injection. Endometrial tissue was injured by a blunt needle-tip syringe that entered the uterine cavity and pulls back and forth twice along the uterine wall. At the same time, 0.1 mL of the prepared multi-pathogen solution which was composed of *S. aureus* and *E. coli* (1 × 10^8^ ccu/mL, respectively) was injected into the ovary, and the rats were positioned upside down for 5 min. Rats in control group were treated identically with saline solution without endometrial tissue injured.

### Histopathological analysis of PID model rats

Ten days after establishment of PID model, the uterine tissue samples of model and normal rats (n = 6 per group) were randomly collected and fixed in 4% paraformaldehyde, followed by embedded in paraffin, cut into 5-μm sections (RM2235, Leica, Germany). Furthermore, paraffin sections were deparaffinized in xylene, then stained with hematoxylin and eosin (H&E) and the pathological changes were evaluated randomly, nonconsecutive chosen fields at a magnification of 200× using optical microscopy (CX21FS1, Olmpus, Japan).

### Drug administration and sample collection

After histological analysis, PID rats were randomly divided into model group, azithromycin group (AZM, 10 mg/kg), high-dose of Fukeqianjin formula group (FF-H, 1.6 g/kg) and low-dose of Fukeqianjin formula group (FF-L, 0.8 g/kg). All drugs were dissolved in distilled water. Normal control group were given equal volume of distilled water. Each group was consisted of 10 rats. After 14 days of intragastric administration, blood samples were collected from abdominal aorta. The plasma samples were centrifuged at 3500 rpm for 10 min at 4 °C and immediately stored at − 80 °C until analysis.

### Measurement of inflammatory cytokines

Concentrations of interleukin-1β (IL-1β) was measured by enzyme linked immunosorbent assay (ELISA) kits (Batch Number: 2301B61242, MultiSciences Biotech Co., Ltd. Hangzhou, China). The concentration of nitric oxide (NO) was quantified based on the nitric reductase method (Batch Number: 20161114, Nanjing Jiancheng Institute of Biotechnology, Nanjing, China). All date was carried out by Varioskan Multifunctional full wavelength microplate reader (Thermo Fisher Scientific, USA) according to the manufacturers.

### Plasm sample preparation

A total of 300 μL acetonitrile was added into 100 μL of plasm sample for protein precipitation, and then the mixture was vortexed for 3 min, centrifuged at 12,000 rpm for 10 min at 4 °C. The supernatant was transferred to an auto-sampler vial for UPLC-Q-Exactive Orbitrap-MS analysis.

### UPLC-Q-Exactive Orbitrap-MS analysis

Chromatographic separation was carried out on an Ultimate-3000 RS LC system (Dionex, USA) using an Acquity UPLC BEH C18 column (2.1 mm × 100 mm, 1.7 μm, Waters, USA) with the column temperature maintained at 35 °C. The mobile phase consisted of 0.1% formic acid in water (A) and 0.1% formic acid in acetonitrile (B). The gradient elution was modified as follows: 0–25 min, 5–95% B; 25–30 min, 95% B; 30–31 min, 95–5% B; and 31–35 min, 5% B. The sample injection volume was set at 10 μL for analysis with a flow rate of 0.4 mL/min.

Mass spectrometry was performed on a Q-Exactive Orbitrap-MS system (Thermo Fisher Scientific, USA) equipped with an electrospray ionization source operating in the positive ion mode with the following parameters: scan type, full MS; scan range, 80 to 1000 m/z; scan resolution, 7000 m/z/s; sheath gas flow rate, 30 arb; aux gas flow rate, 10 arb; spray voltage, 3.5 kV, capillary temperature, 320 °C; aux gas heater temperature, 150 °C.

The quality control (QC) samples mixed by 100 μL of 10 experimental samples were determined by duplicate analysis of six injections before analysis to evaluate the precision and repeatability of the instrument. In addition, QC samples were measured every 10 samples during the testing process in order to investigate the stability of analytical method by determining relative standard deviations (RSD) of intensity and retention time of 10 randomly selected characteristic ion peaks of QC samples.

### Data analysis

The raw MS data were exported to MZ mine for normalization treatment before multivariate analysis. The principal component analysis (PCA), partial least-squares discriminant analysis (PLS-DA) and orthogonal partial least-squares discriminant analysis (OPLS-DA) were performed with Metabolomics Univariate and Multivariate Analysis (MUMA) of R software (URL: http://www.R-project.org/). For potential biomarker identifications, the information was obtained from the following databases: http://www.genome.jp/kegg/, http://metlin.scripps.edu/, http://www.lipidmaps.org/. Potential metabolic pathways were analyzed by Metabo Analyst 2.0. All results were expressed as the mean ± standard deviation (SD). One-way analysis of variance (ANOVA) was applied to the statistical analysis and P values less than 0.05 were considered significantly different between groups.

## Results

### Quality control of Fukeqianjin formula

**Fig. 1:**
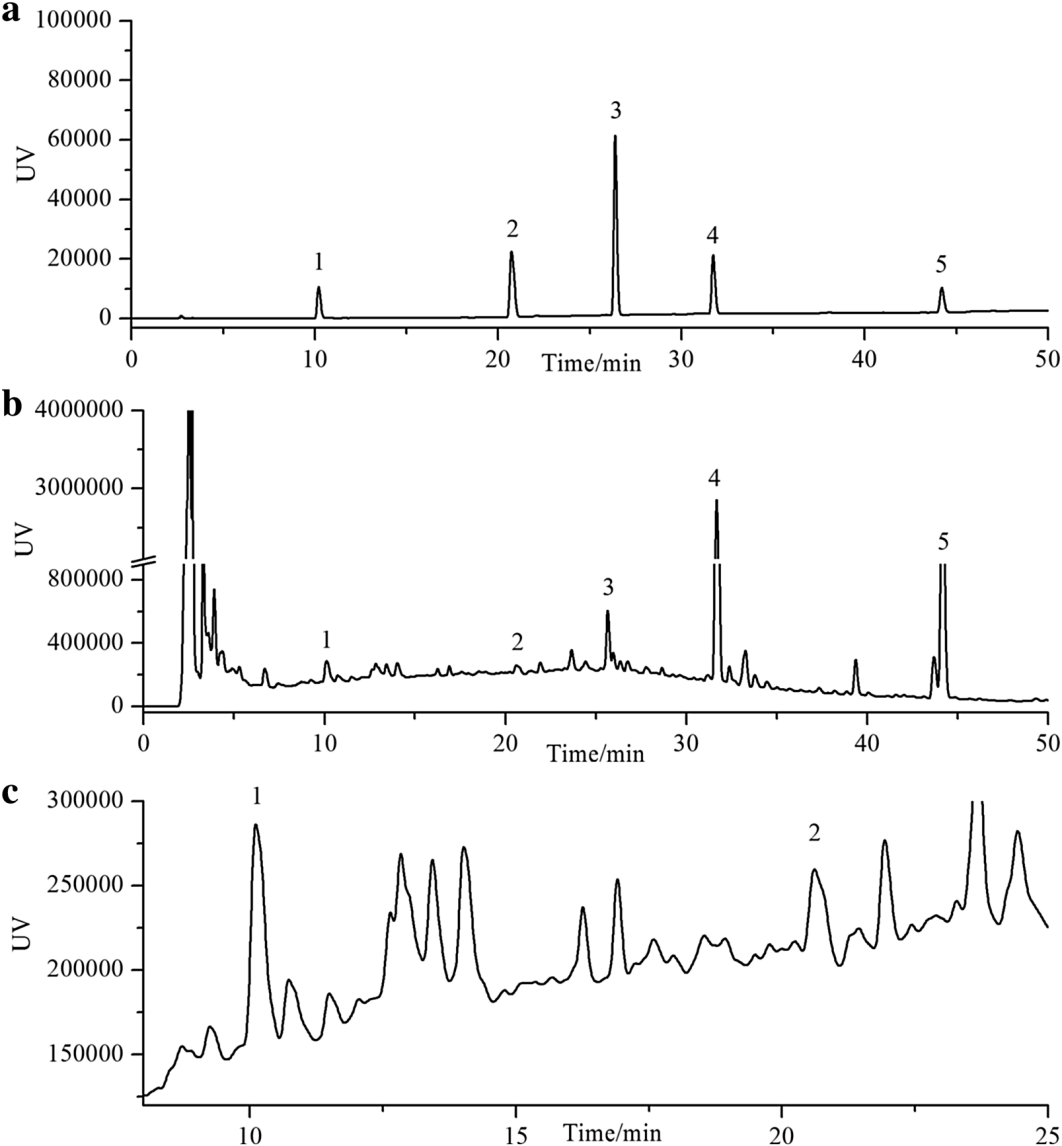
The typical HPLC chromatogram of Fukeqianjin formula. **a** Mixed standard reference substance. 1: chlorogenic acid (10.163 min); 2: ferulic acid (20.775 min); 3: lobetyolin (26.385 min); 4: andrographolide (31.671 min); 5: dehydrated andrographolide (44.164 min). **b** Fukeqianjin formula extract (0–50 min). **c** Fukeqianjin formula extract (8–25 min)

Five major characteristic chemical constituents from Fukeqianjin formula were determined. A typical HPLC chromatogram was shown in Fig. 1. As a result, the concentration of chlorogenic acid, ferulic acid, lobetyolin, andrographolide and dehydrated andrographolide was 0.46 mg/g, 0.33 mg/g, 0.25 mg/g, 2.06 mg/g, and 1.67 mg/g, respectively. Fukeqianjin formula conform to the relevant quality standards.

### Histopathological examination of PID model

**Fig. 2:**
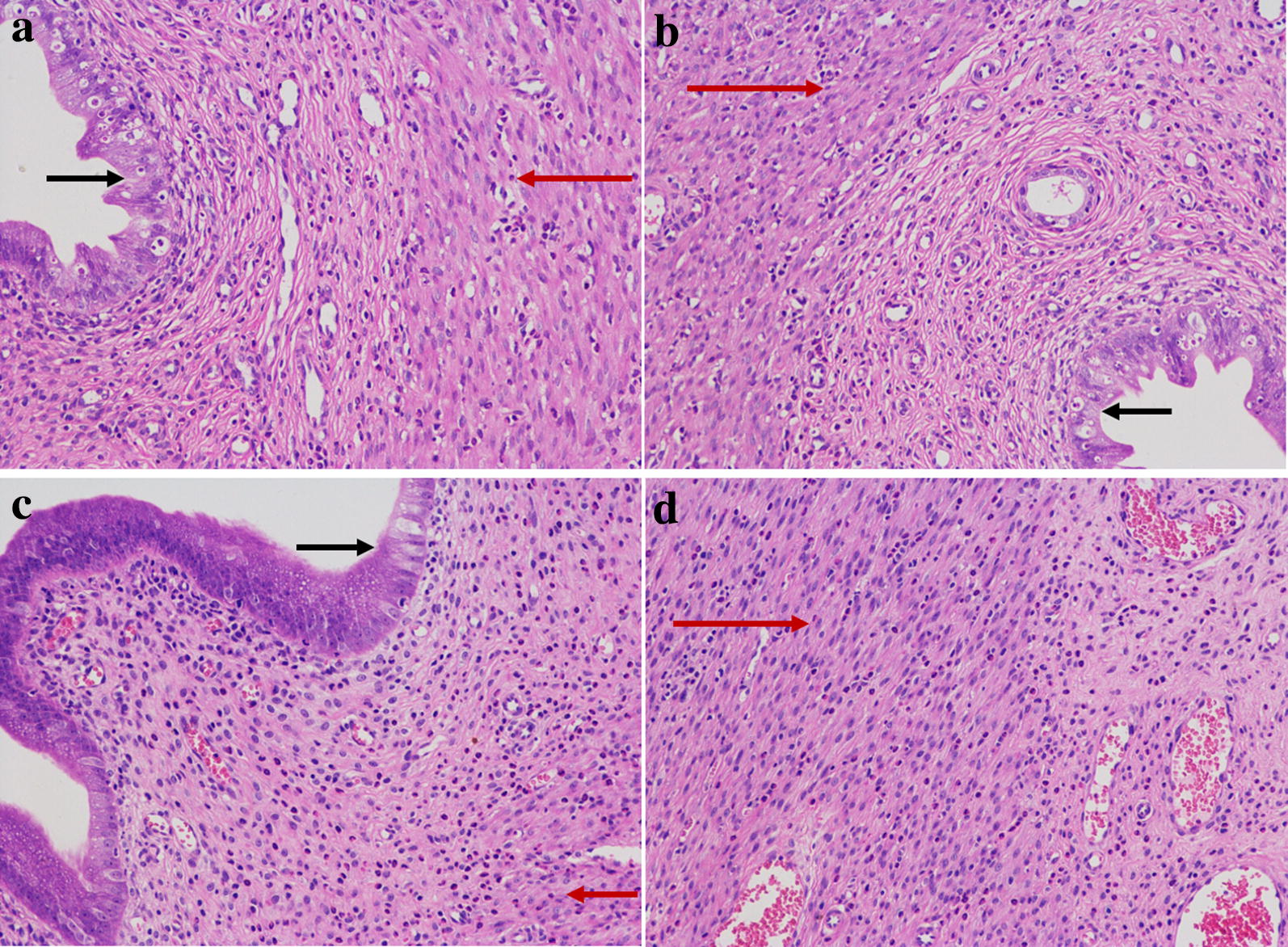
Representative histopathological micrographs of uterus. **a, b** Saline solution treatment. **c, d** Multi-pathogen solution treatment. H&E stain, ×200. Black arrow stands for endometrium, red arrow stands for myometrium

In PID group, the uterus of rats were characterized by obvious congestion and edema, epithelial cell proliferation and degeneration, and chronic inflammatory cell infiltration was observed in the endometrium and myometrium. It indicated that the multi-pathogen treatment with endometrial tissue injure could result in the inflammation reaction in the upper genital tract (Fig. 2).

### Anti-inflammation activity

**Fig. 3:**
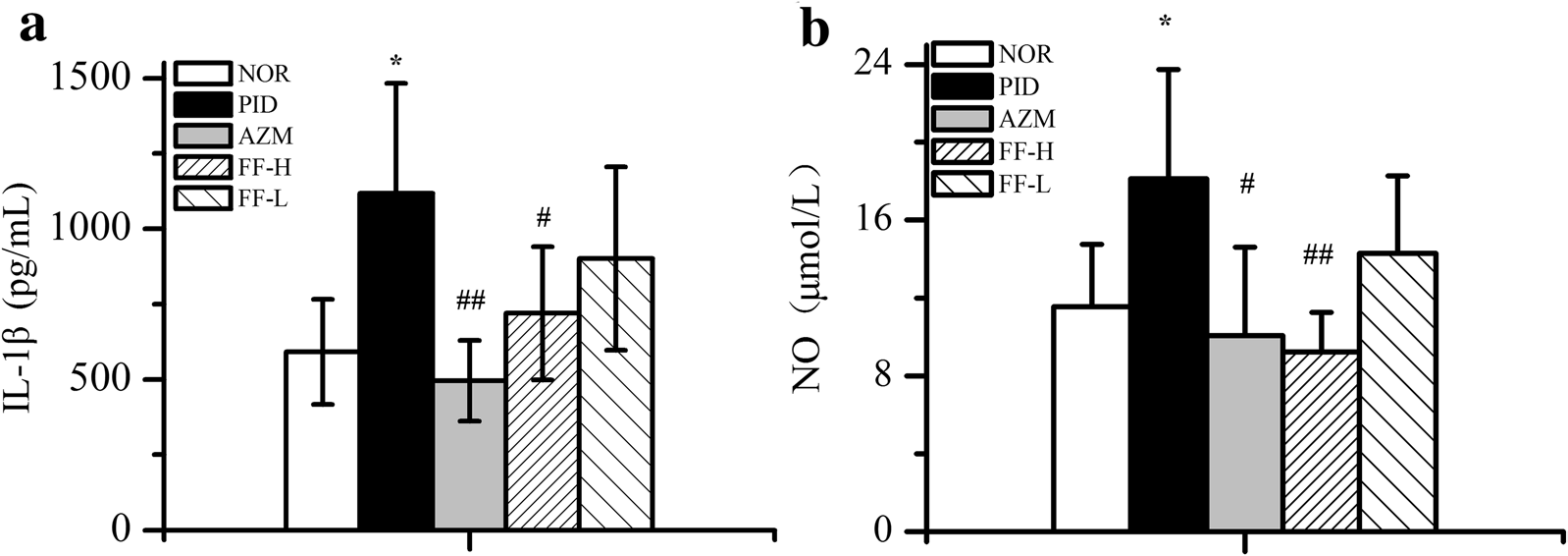
Effect of Fukeqianjin formula on the inflammatory response in PID rats. **a** Level of IL-1β. **b** Level of NO. *P < 0.05 compared to the control group; ^#^*P* < 0.05 and ^##^*P* < 0.01 compared to the PID model group

As illustrated in Fig. 3, compared with normal control group, the inflammatory cytokines including IL-1β and NO were significantly increased in PID rats. Treatment with either AZM or FF-H could remarkably decrease the level of IL-1β and NO in plasma, which can be inferred that AZM and FF-H can alleviate genital inflammatory response in rats with pelvic inflammation.

### Validation of UPLC-Q-Exactive Orbitrap-MS conditions

**Fig. 4:**
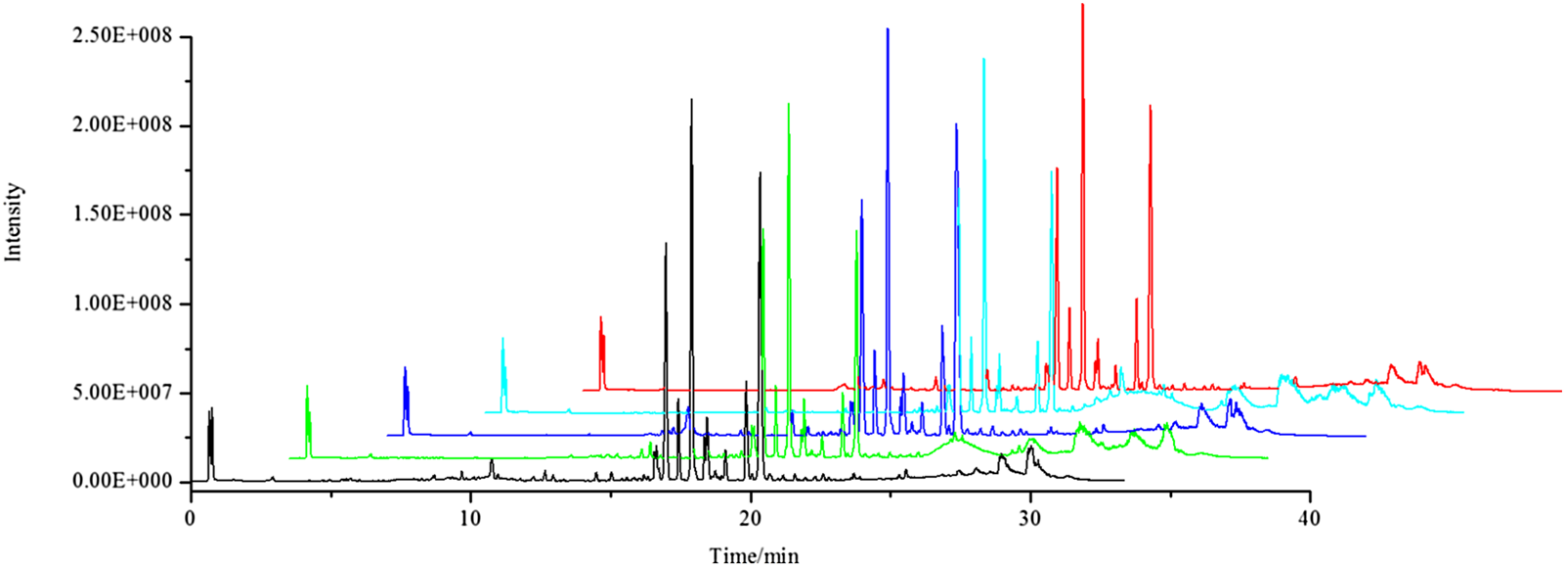
Representative UPLC-Q-Exactive Orbitrap-MS TIC chromatograms of the plasma samples. Black line: NOR group, Green line: AZM group, Blue line: FF-H group, Light blue line: FF-L group, Red line: PID group

The representative UPLC-Q-Exactive Orbitrap-MS total ion chromatogram (TIC) of the plasma samples were shown in Fig. 4. By calculating the peak area, m/z data, and retention time of the selected ions, the precision, repeatability of the instrument, and stability of analytical method were evaluated. All of the relative standard deviation (RSD%) values were less than 6%, which indicated that this established rat plasma metabolomics analysis method was reliable and accurate.

### Pattern recognition and potential biomarkers identification

**Fig. 5:**
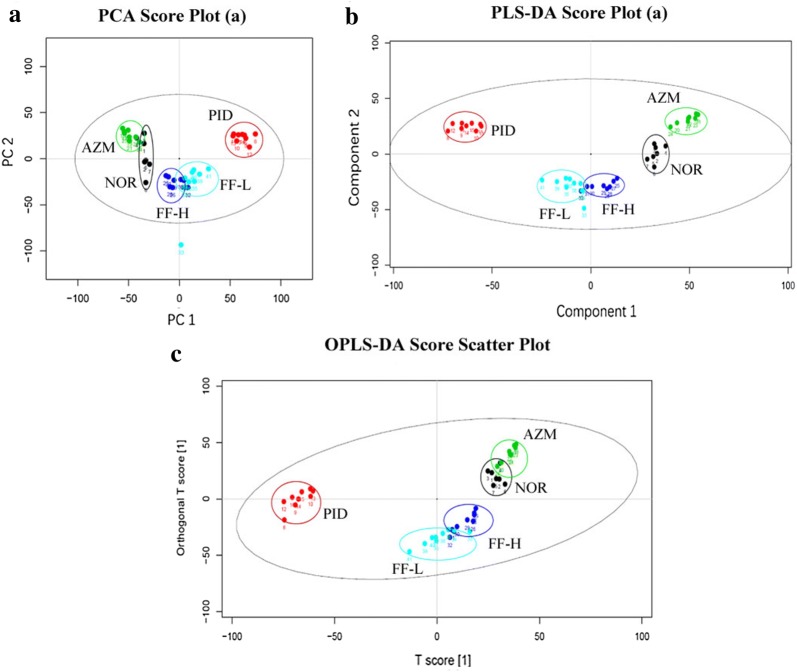
Scores plots of PCA (**a**), PLS-DA (**b**) and OPLS-DA (**c**) analysis on the PID rat plasm metabolic profiles of normal control (NOR), model (PID), azithromycin treatment (AZM) and Fukeqianjin formula treatment (FF-H, FF-L) group

First, PCA analysis was used for unsupervised pattern recognition in all groups. Additionally, two supervised pattern recognition methods, PLS-DA and OPLS-DA, were employed to provide better discrimination and to carry out metabolites differences between groups in this study. As shown in Fig. 5, the corresponding scores plots from PCA, PLS-DA and OPLS-DA showed that PID rats were clearly separated from normal rats, which suggested that intrauterine inoculation of multiple pathogens combined with mechanical injury of endometrium could significantly disturb the plasma metabolic profiles of rats. Meanwhile, compared with the PID model group, the metabolite profiles of Fukeqianjin formula treatment group was gradually restored to normal, indicating that the treatment of Fukeqianjin formula can significantly alleviate the metabolic disorders caused by pelvic inflammation in rats.

**Fig. 6:**
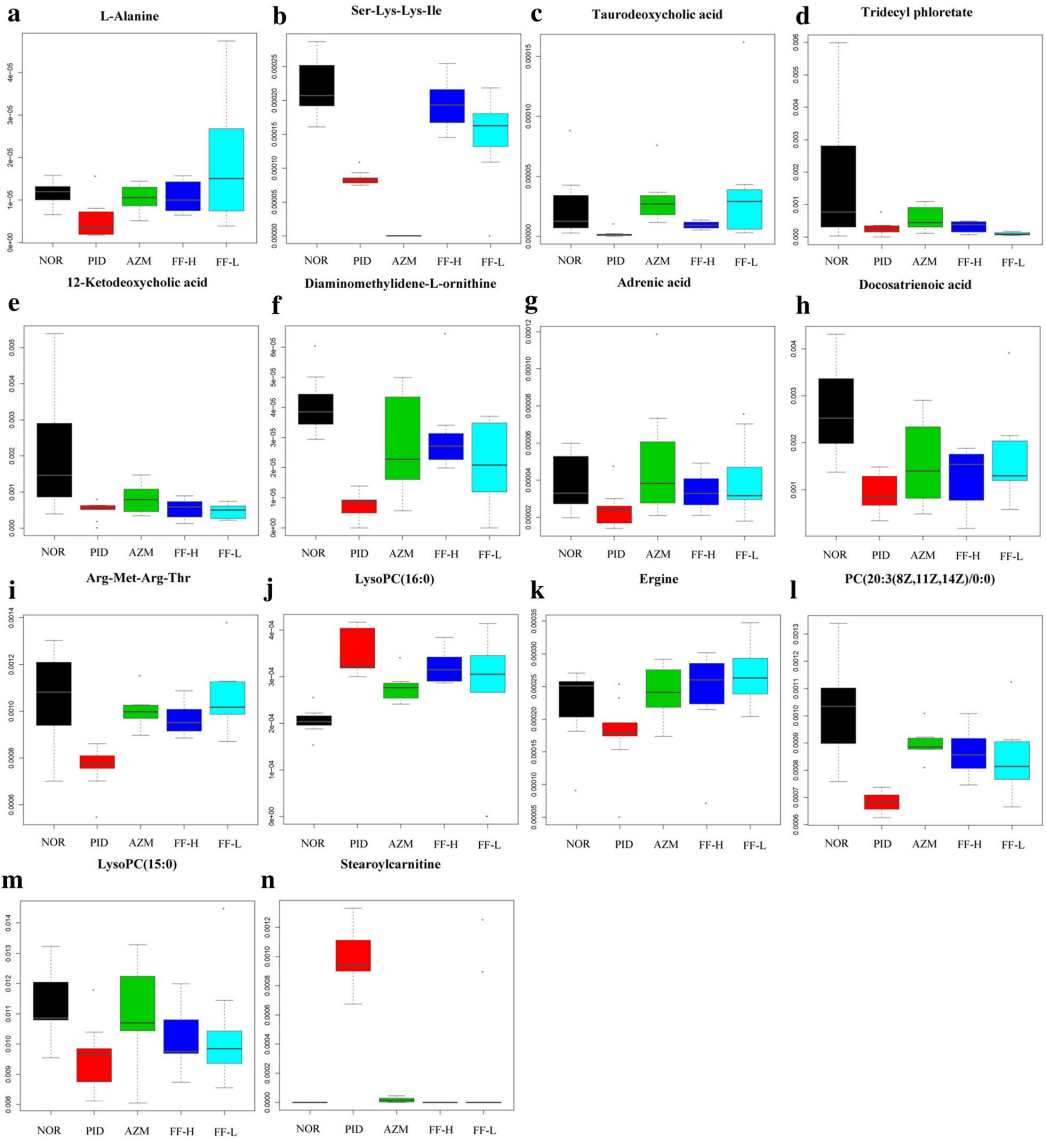
Boxplots of identical biomarkers normalized chromatography peak intensity of rat plasma

A total of 14 potential metabolite biomarkers, including l-alanine, Ser-Lys-Lys-Ile, taurodeoxycholic acid, tridecyl phloretate, 12-ketodeoxycholic acid, diaminomethylidene-l-ornithine, adrenic acid, docosatrienoic acid, Arg-Met-Arg-Thr, Ergine, PC(20:3(8Z,11Z,14Z)/0:0), LysoPC(15:0), LysoPC(16:0), and stearoylcarnitine, which are mainly related to the metabolic pathways of intervening glycerophospholipid metabolism, linoleic acid metabolism/alpha-linolenic acid metabolism, amino acid metabolism, arachidonic acid metabolism, and unsaturated fatty acids biosynthesis were identified (shown in Table 1). The different intensity of these biomarkers was shown in Fig. 6. The results also suggested that the plasma biomarkers of rats with pelvic inflammation have obvious changes compared with normal rats, and Fukeqianjin formula has potential therapeutic effect on PID rats.

**Table 1:** The potential metabolite biomarkers

No.	RT (min)	Mass (m/z)	Formula	Identity names	Trend
1	0.74	90.0544	C_3_H_7_NO_2_	l-Alanine	↓
2	5.67	475.3232	C_21_H_42_N_6_O_6_	Ser-Lys-Lys-Ile	↓
3	10.06	500.3023	C_26_H_45_NO_6_S	Taurodeoxycholic acid	↓
4	11.37	357.2775	C_22_H_36_O_3_	Tridecyl phloretate	↓
5	11.39	391.2826	C_24_H_38_O_4_	12-Ketodeoxycholic acid	↓
6	11.54	750.3722	C_28_H_51_N_11_O_13_	Diaminomethylidene-l-ornithine	↓
7	12.61	355.2618	C_22_H_36_O_2_	Adrenic acid	↓
8	15.34	357.2775	C_22_H_38_O_2_	Docosatrienoic acid	↓
9	16.99	563.3104	C_21_H_42_N_10_O_6_S	Arg-Met-Arg-Thr	↓
10	17.13	496.3379	C_24_H_50_NO_7_P	LysoPC (16:0)	↑
11	17.41	268.1477	C_16_H_17_N_3_O	Ergine	↓
12	17.78	546.3533	C_28_H_52_NO_7_P	PC (20:3(8Z,11Z,14Z)/0:0)	↓
13	20.18	482.3223	C_23_H_48_NO_7_P	LysoPC (15:0)	↓
14	22.91	428.3715	C_25_H_49_NO_4_	Stearoylcarnitine	↑

## Discussion

Nitric oxide (NO), an important endogenous bioinformatic molecule produced from l-arginine by constitutive and inducible nitric oxide synthases, plays a key role in inflammatory cascade reaction and immune regulation [24, 25]. Inhibiting the excessive production of NO has been regarded as an effective strategy for the treatment of inflammation-related diseases due to its harmful consequences such as tissue damage in the process of chronic inflammation [26]. It was previously reported that stimulating IL-1β would increase the expression of iNOS, which may further cause an increased production of NO [27]. Study indicated the amount of NO in endometrial tissues was comparatively higher in patients with endometriosis [28]. Rocha et al. reported that NO levels appeared to be elevated in women with chronic pelvic pain diagnosed as secondary stage to endometriosis, and was directly associated with reduction in pain intensity and increase in pain threshold after treatment [29]. Meanwhile, recent experiments have shown that the productions of inflammatory cytokines including IL-1β, IL-6, and TNF-α were significantly increased in upper genital tract of PID rats [16, 17]. In patients with upper genital tract inflammatory diseases, the level of interleukins IL-1β were prominently elevated [30]. In this study, NO and IL-1β level in PID rat plasma was detected to evaluate the anti-inflammatory function of Fukeqianjin formula. Results show that the level of NO and IL-1β were significantly increased (Fig. 3), indicating inflammatory response has been induced in PID rats. Moreover, treatment with FF-H remarkably decrease these cytokines levels, suggesting a potent anti-inflammatory effect of FF-H.

A systematic review and meta-analysis drew a conclusion that *A. paniculata*, an important drug in Fukeqianjin formula, was safe and effective in the treatment of acute respiratory tract infections [31]. Andrographolide is the main chemical constituent of *A. paniculata* and is also considered to be a major contributor to the therapeutic activity [32, 33]. It has been reported that andrographolide has a broad-spectrum antimicrobial activity against both Gram (+) and Gram (−), especially to *S. aureus*, *Bacillus subtilis*, *E. coli*, *Pseudomonas aeruginosa*, and *Klebsiella pneumoniae* [34]. In addition, andrographolide was able to inhibit *Chlamydia trachomatis*-induced human cervical epithelial cell infection and reduce the level of inflammatory factors such as IFNγ-induced protein10, CXCL8, and IL-6 [35]. Study also confirmed that andrographolide administration in both LPS-activated RAW264.7 cells and peritoneal macrophages could decrease IFN-β, iNOS, TNF-α, and COX-2 expression as well as the downstream NO and PGE2 productions [36]. *Andrographis paniculata* has also been proved to have potent therapeutic effect on pathogen-induced PID rats by significantly reduce the excessive secretion of cytokines and chemokines including IL-1β, IL-6, CXCL-1, and MCP-1 through blocking the NF-κB signal pathway transduction [37]. Based on our experimental results and previous studies, we speculate that *A. paniculata* may be one of the main anti-inflammatory drugs in Fukeqianjin formula.

Besides, it was previously reported that the total flavonoids from *R. laevigata Michx* fruit markedly downregulated the expression levels of IL-1β, IL-6, TNF-α by inhibiting NF-κB and AP-1 transcriptional activities during inflammatory courses, and thus has protective effect on pathological processes including renal and hepatic ischemia–reperfusion damage [38, 39]. Berberine, as the main ingredient of *Mahonia fortunei*, was widely recognized for its considerable anti-inflammatory effects. Study has demonstrated that berberine has a protective effect on adenomyosis, a complication of PID, by inhibiting the expression of IL-6, IL-8, TGF-β, VEGF and MMP-2 [40]. Its anti-inflammatory mechanism mainly involves signaling pathways such as NF-κ B, AMPK, and caspase-1/IL-1β inflammatory signal transduction axis [41, 42]. Combination of the above herbal medicine can enhance the anti-inflammatory effect of the compound, and produce a good therapeutic effect on pelvic inflammation.

At the same time, although PID is recognized as a multiple bacterial infectious disease, chronic inflammation and correlative complications also participate in its progression. So, in addition to short-term antimicrobial targets, long-term sequelae prevention and treatment is also an important therapeutic goal [43]. Some studies indicates that chronic PID are often under a situation of microcirculation disorder and immune deficiency. *Angelica sinensis* is commonly used in Chinese medicine to promote hematopoietic function, improve microcirculation and regulate the immune system [44]. Ferulic acid, Z-ligustilide and polysaccharides are the main chemical constituents of *Angelica sinensis* [45]. Liu et al. reported polysaccharides may promote the recovery of platelets and other blood cells and the formation of hematopoiesis in rat by regulating the PI3K/Akt pathway [46]. *Spatholobus suberectus* contains various types of polyphenolic compounds mainly including flavonoids, isoflavones, flavonols, and flavanones [47]. Among them, total flavonoids of *S. suberectus* have been proved to inhibit oxidative stress and regulate immune function, and it can also play an anti-viral role [48]. Through reasonable compatibility, the whole compound can not only achieve good anti-inflammatory effect, but also improve microcirculation and immunity. It has significant advantages in the treatment of pelvic inflammatory disease and its complications.

In addition, since the complex composition of Chinese herbal compound, it is difficult to elucidate its mechanism of action. Metabonomics methodology covers a wide range of substances, focusing on holistic and dynamic evaluation, which was in coincidence with the holistic theory of traditional Chinese medicine. In recent years, metabonomics has been widely used in the study of inflammatory diseases. Previous studies have shown that collagen-induced arthritis was characterized by metabolic disorders of lipid, tricarboxylic acid cycle, tryptophan and phenylalanine metabolism [49]. According to Ahn et al. the mechanism of curcumin in the treatment of rheumatoid arthritis may be related to the abnormal regulation of glycine, citrulline, arachidonic acid and saturated fatty acid levels [50]. In this study, the plasma metabolomics method based on UPLC-Q-Exactive Orbitrap-MS was used to investigate the underlying mechanism of Fukeqianjin formula on pelvic inflammation. Our results demonstrate that 14 endogenous compounds can be considered as potential metabolite biomarkers of PID (Table 1). Studies have confirmed that the metabolites including amino acids, fatty acids, organic acids, and sugars in urine could considered to be the potential biomarkers of PID [22]. In addition, *Patrinia scabiosaefolia Fisch* is believed to play a therapeutic role in pelvic inflammation by regulating tricarboxylic acid circulation, glucose metabolism, and amino acid metabolism [23]. However, the above studies were based on the urine metabolomics of gas chromatography–mass spectrometry and our study can be used as complementary biological information for PID.

Previous studies suggested that the lipids metabolism, including bile acids and unsaturated fatty acids, is involved in the occurrence and development of inflammatory reaction [51]. Taurine deoxycholic acid has been shown to ameliorate insulin resistance in mice by inhibiting the increase of phospholipid, myelin sheath and neuramide [52]. Moreover, taurine deoxycholic acid can significantly up-regulate the expression of MUC4 (a membrane-bound mucin) by activating phosphatidylinositol 3-kinase, thereby affecting cell proliferation and tumor progression [53, 54]. As a long chain polyunsaturated fatty acids, docosatrienoic acid is of great benefit to health. As a metabolite of arachidonic acid, adrenic acid can be converted into a variety of oxygenated metabolites, and plays an important role in cardiovascular system and immune system [55]. In this study, a variety of bile acids and unsaturated fatty acids, including taurodeoxycholic acid, 12-ketodeoxycholic acid, docosatrienoic acid and adrenic acid, were identified (Table 1). After treatment with Fukeqianjin formula for 14 days can significantly correct the abnormal changes of these substances (Fig. 6).

Studies have shown that some kinds of phosphatidylcholine (PC) were important biomarkers of inflammation. Glycerophospholipids metabolism profiles can characterize the progress of inflammation and provide valuable evidence for the diagnosis and prognosis of inflammation [56]. It has been confirmed that various drugs in Fukeqianjin formula can affect lipid metabolism. For instance, the total flavonoids of *R. laevigata* Michx can regulate lipid metabolism in LPS-induced liver injury mice by mainly reducing the expression levels of fatty acid synthase, acetyl coenzyme A carboxylase-1, and stearoyl coenzyme A desaturase-1, and improving the level of carnitine palmitoyltransferase 1 [57]. Besides, some new polyynes from *Codonopsis pilosula* may affect lipid metabolism by inhibiting the expression of squalene monooxygenase gene in HepG2 cells [58]. Our data suggested that there were significant changes in biomarkers, including LysoPC (16:0), PC (20:3(8Z,11Z,14Z)/0:0), and LysoPC (15:0), in PID rats, which could be regulated by Fukeqianjin formula intervention (Fig. 6).

Amino acids are not only essential substances of proteins, but also as signal molecules regulate various physiological functions of the organism, such as precursors of many neurotransmitters and hormones, and intermediates of TCA circulation and glyconeogenesis [59]. Previous reports have demonstrated that branched-chain amino acids can increase the expression of eNOS and nitrotyrosine, and induce inflammatory response by activating the transcription factor NF-κB in endothelial cells [60]. Four potential biomarkers related to amino acid metabolism, including l-alanine, Ser-Lys-Lys-Ile, diaminomethylidene-l-ornithine, and Arg-Met-Arg-Thr, have been shown in Table 1. Fukeqianjin formula was observed to have an obvious regulatory effect on the abnormal changes of these substances (Fig. 6). To summarize, the results illustrated that the metabolic pathways were mainly related to glycerophospholipid metabolism, linoleic acid metabolism/alpha-linolenic acid metabolism, amino acid metabolism, arachidonic acid metabolism, and unsaturated fatty acids biosynthesis. Fukeqianjin formula has potential therapeutic effect on multi-pathogen-induced PID rats by ameliorating metabolism disorders.

## Conclusions

In summary, these results indicate that intrauterine inoculation of multiple pathogens combined with mechanical injury of endometrium could significantly disturb the plasma metabolic profiles of rats. Fukeqianjin formula has potential therapeutic effect on multi-pathogen-induced PID rats by ameliorating metabolism disorders and alleviating the inflammatory response.

## Additional file

**Additional file 1.** The minimum standards of reporting checklist.

## Abbreviations

PID: pelvic inflammatory disease
FF: Fukeqianjin formula
AZM: azithromycin
IL-1β: interleukin-1β
IL-8: interleukin-8
IL-10: interleukin-10
IL-2: interleukin-2
NO: nitric oxide
TNF-α: tumor necrosis factor-α
NF-κB: nuclear factor-κb
IgA: immunoglobulin A
IgG: immunoglobulin G
IgM: immunoglobulin M
UPLC-Q-Exactive Orbitrap-MS: ultra-performance liquid chromatography-quadrupole-Exactive Orbitrap-mass spectrometry
NMR: nuclear magnetic resonance
GC–MS: gas chromatography–mass spectrometer
HPLC: high performance liquid chromatography
PCA: principal component analysis
PLS-DA: partial least squares discriminant analysis
OPLS-DA: orthogonal partial least squares discriminant analysis
CCl4: carbon tetrachloride
H&E: hematoxylin and eosin
QC: quality control
RSD: relative standard deviations
MUMA: Metabolomics Univariate and Multivariate Analysis
SD: standard deviation
ANOVA: one-way analysis of variance
TIC: total ion chromatogram
IFNγ: interferon γ
LPS: lipopolysaccharide
iNOS: inducible nitric oxide synthase
COX-2: cyclooxygenase-2
PGE2: prostaglandin E2
TFs: total flavonoids
VEGF: vascular endothelial growth factor
TGF-β: transforming growth factor-β
AMPK: adenosine 5′-monophosphate (AMP)-activated protein kinase
MMP-2: matrix metalloproteinases-2
